# Driving Performance on the Descending Limb of Blood Alcohol Concentration (BAC) in Undergraduate Students: A Pilot Study

**DOI:** 10.1371/journal.pone.0118348

**Published:** 2015-02-27

**Authors:** Mathieu Tremblay, François Gallant, Martin Lavallière, Martine Chiasson, Dustin Silvey, David Behm, Wayne J. Albert, Michel J. Johnson

**Affiliations:** 1 Faculty of Kinesiology, University of New Brunswick, Fredericton, Canada; 2 École de kinésiologie et de loisir, Université de Moncton, Moncton, Canada; 3 Healthy Driver Research Group, Université de Moncton, Moncton, Canada; 4 MIT (Massachusetts Institute of Technology) AgeLab & New England University Transportation Center, Cambridge, United States of America; 5 School of Human Kinetics and Recreation, Memorial University of Newfoundland, St John’s, Canada; The Scripps Research Institute, UNITED STATES

## Abstract

Young drivers are overrepresented in collisions resulting in fatalities. It is not uncommon for young drivers to socially binge drink and decide to drive a vehicle a few hours after consumption. To better understand the risks that may be associated with this behaviour, the present study has examined the effects of a social drinking bout followed by a simulated drive in undergraduate students on the descending limb of their BAC (blood alcohol concentration) curve. Two groups of eight undergraduate students (n = 16) took part in this study. Participants in the alcohol group were assessed before drinking, then at moderate and low BAC as well as 24 hours post-acute consumption. This group consumed an average of 5.3 ± 1.4 (mean ± SD) drinks in an hour in a social context and were then submitted to a driving and a predicted crash risk assessment. The control group was assessed at the same time points without alcohol intake or social context.; at 8 a.m., noon, 3 p.m. and 8 a.m. the next morning. These multiple time points were used to measure any potential learning effects from the assessment tools (i.e. driving simulator and useful field of view test (UFOV)). Diminished driving performance at moderate BAC was observed with no increases in predicted crash risk. Moderate correlations between driving variables were observed. No association exists between driving variables and UFOV variables. The control group improved measures of selective attention after the third asessement. No learning effect was observed from multiple sessions with the driving simulator. Our results show that a moderate BAC, although legal, increases the risky behaviour. Effects of alcohol expectancy could have been displayed by the experimental group. UFOV measures and predicted crash risk categories were not sentitive enough to predict crash risk for young drivers, even when intoxicated.

## Introduction

Young driver education, training programs, and legislative changes have been successful at decreasing the number of collisions over the last few decades [1–3]. Over the last few years, in Canada, many provinces (that enforce their own driving legislation) have put in place restrictive measures for young drivers. For instance, in New Brunswick (Canadian province on the east coast), since 2009 individuals between the ages of 16 and 21 years must go through a graduated licensing program to obtain a driving permit. They cannot operate motor vehicles between midnight and 5 a.m. There is zero tolerance for alcohol, therefore drivers must have a blood alcohol concentration (BAC) of 0.0 g/dL. Individuals who carry a learner’s permit can have no more than one teenage passenger at a time. After successfully finishing the graduated licensing program (without violations), drivers 21 years of age become unrestricted, meaning that the zero alcohol tolerance policy is lifted. Despite all actions taken, young drivers remain the most at-risk group of drivers, underscoring the need to further understand the factors underlying collisions in this population.

In 2010, 27.7% of alcohol-related crashes resulting in serious injuries involved drivers between the ages of 16 and 25 years [4]. Overall, this age group was implicated in 33.2% of all serious alcohol-related injuries in Canada that year [4]. More alarming, the number of deaths in this age group was 593, with alcohol being a factor in 299 of these cases, representing 50% of all deaths in this age group [4]. The drivers of this age are scattered between the multiple steps of the licensing program. Even though this age group represents only 13.6% of the Canadian population, it is responsible for 33.3% of alcohol-related crashes resulting in fatalities [5].

Many factors contribute to collisions in younger drivers, including driver inexperience, risky driving behaviour, and drug and/or alcohol consumption [6,7]. By far, alcohol is one of the most dangerous yet preventable factors responsible for driving-related collisions. Alcohol can play an important role in many social events for this age group, and the acute impact of alcohol consumption on driving is widely studied [8]. However, it is not uncommon for individuals to consume large amounts of alcohol in a short period of time, and to then take the wheel a few hours later when they have reached legally acceptable BAC levels. A recent epidemiological study by French & Gumus [9], observed not only a significant increased prevalence of motor vehicle collision during the spring break season involving young drivers, but also a significant number of these crashes involving drivers with a BAC below 0.08 g/dL. This level is not considered legally impaired in North America. Although there is a preponderance of studies examining the effects of alcohol consumption on performance shortly following intoxication, there are a lack of studies examining driving performance after a more prolonged abstinence.

To better understand the effects of common practices regarding alcohol consumption observed in young drivers and driving performance, the present pilot study examined the effects and relationships between simulated driving performance and crash risk in young drivers with BAC decreasing over time. This study recreated a combined social gathering and impaired driving context, while capturing direct measures of driving performance.

## Materials and Methods

### Design

An experimental design with two groups of young drivers was chosen for observation: 1) the effects of decreasing BAC on driving performance and crash risk in the first group; and 2) the second group was used to control potential learning effects (performance improvement) of multiple sessions with assessment tools (i.e. driving simulator and useful field of view (UFOV)). For this pilot study, the statistical power was not enough (i.e. < 0.95; α = 0.05) to be able to conduct between-group analysis. Therefore, strictly intra-group comparisons were performed.

### Participants

Two groups of 10 participants (12 women, 8 men) volunteered to take part in this study. Of the twenty participants, four participants were unable to complete the protocol (two in each group) due to simulator sickness. To be eligible, participants were required to have a valid driver’s license and be at least 19 years old, the legal drinking age in the province of New Brunswick. Participants were excluded from the study if they had chronic diseases, had issues linked to alcohol consumption or did not consume alcohol weekly (at least 1 drink par week). Pregnancy was also an exclusion factor. All drivers were recruited from the academic community of the university. This experiment was specifically approved by the *Comité d'éthique de la recherche avec les êtres humains* of the *Université de Moncton* (reference number is CER-1213032).

Experimental group: The participants were invited to binge drink in a social gathering event that took place in a controlled environment in the lab, and were encouraged to invite a friend to this event. All participants were advised to abstain from drinking alcohol 24 hours prior to testing, have a good night’s sleep and eat well before the experiment. This group had an average age of 21.6 ± 2.32 years (2 women, 6 men) and consumed an average of 7 ± 4.6 drinks weekly. For safety while consuming alcohol, a registered nurse supervised the participants.

Control group: In this group, participants completed the same protocol but without consuming alcohol. This group had a mean age of 20.9 ± 2.35 years (4 women, 4 men). Since this group did not consume alcohol during this study, alcohol related information was not recorded (i.e. issues with alcohol consumption, consumption habits).

### Apparatus

Physiological assessment: A 3-lead ECG (MLA2340), was used to collect, condition (i.e. amplification, filtering, converting) and record heart signals with the help of the Bio Amp unit (FE132) and an eight channel PowerLab unit (PL3508) (AdInstruments, United States of America (USA)). LabChart software (version 7, AdInstruments) was used for data collection, data analysis and calculation of heart rate variability (HRV). HRV was used to measure the physiological response to alcohol [10,11].

Crash risk prediction: The Useful Field of View (UFOV) test is one of the most widely used and better predictors available of driving performance and crash risk [12,13]. It is comprised of three subtests measuring: 1) processing speed; 2) divided attention; and 3) selective attention. These represent higher-order cognitive functioning required for safe vehicle driving. After completing all three subtests, the software places each participant into a relative crash risk category ranging from very low to very high. This test was performed on a 17” touch screen (Elo Touchsystems 2700 Intellitouch USB) with UFOV software (version 6.1.4; Visual Awareness Research Group inc., USA).

Driving simulator: The simulated drives were completed on a driving simulator (VS500M, Virage Simulation, Canada). The open car simulator resembles a General Motors (GM) compact cab interior. The simulator consists of a driver’s seat, steering column, pedals, automatic transmission and a dashboard, which are mounted on a three-axis motion/vibration platform that provides force feedback and vibration. Three 52” LCD displays provide 180 degrees front view. The resolution is 1920x1080 pixels per front display. Rear view and side view mirrors are simulated through the screens. Two side screens located behind the driver provide additional visual feedback for the left and right blind spots.

### Procedures

Upon arrival at the laboratory, each participant was briefed on the procedures of the experiment. All participants read and signed a consent form approved by the research ethics committee of the *Université de Moncton* (CER-1213032). Participants were then given a general demographic questionnaire. In addition, a registered nurse sat with each participant and asked questions related to alcohol consumption and alcohol behaviour. Following this initial screening, 3-lead ECG electrodes were placed on the participants. Next, the participants were guided through a 10 minute practice driving scenario to get familiarized with the simulator. After this adaptation period, a 5 minute rest period was provided before proceeding with the UFOV and in-simulator driving assessment.

The simulated driving scenario was an urban drive that took an average of 13.6 ± 1.41 minutes. This drive occurred on a clear day through a city while encountering different levels of traffic density with other road users. Participants were guided through the simulation using a programmed voice (similar to a car GPS navigator) instructing participants to turn left or right at various intersections. All participants underwent the same driving scenario. Both groups were assessed at four time points (alcohol conditions):

- 8 a.m. on the first assessment day (BAC at 0 g/dL; Sober),
- 12 p.m. (four hours (4) after acute consumption with BAC within 0.05–0.07 g/dL; Moderate BAC),
- 3 p.m. (seven hours (7) after acute consumption with BAC within 0.01–0.04 g/dL; Low BAC),
- 8 a.m. the next morning (twenty-four hours (24) after acute consumption with BAC at 0 g/dL; 24H).

The participants in the control group were assessed at corresponding times of day (8 a.m., noon, 3 p.m. and 8 a.m. the next morning). To validate that the experimental participants did not exceed target BAC values, an ethylometer (Dräger Alcotest 7410 GLC, Draeger Safety Canada Ltd, Canada) was used before each testing period. All participants were at the appropriate BAC values before and during testing. This ethylometer was a loan from the city police service. Participants in the alcohol group were asked to consume one drink (30 mL of 40% Vodka) for every 11 kg of body weight in 60 minutes. The total number of drinks to be consumed was equally distributed over the hour. The average consumption of 30 mL drinks in one hour was 5.3 ± 1.4. This quantity of alcohol was deemed necessary to bring participants’ BAC over 0.10 g/dL (high BAC), similar to what could be expected during a binge-drinking scenario. Following consumption, the estimation of BAC was calculated with a Canadian adaptation of the Widmark formula [14], where a standard drink contains 13.6 g of alcohol [15]. This formula was used to define the amount of time required for BACs to be at moderate and low levels. Widmark formula:

BAC=(0.806*SD*1.36)/(BW*Wt)−(MR*DP)

where, 0.806 is a constant for body water in the blood (mean 80.6%), SD is the number of standard drinks (10 g ethanol per drink); 1.36 is the factor to convert the amount in grams to Canadian standards, BW is the body water constant (0.49 for females, 0.58 for males); Wt is body weight (kg); MR is metabolism rate of alcohol elimination (0.017 for females, 0.015 for males); and DP is the drinking period (hour).

### Dependant variables

#### HRV

- Mean heart rate (MHR) in beats per minute (bpm).
- Mean time intervals between normal-to-normal beats (MeanNN) in milliseconds (ms).
- Standard deviation time intervals between normal-to-normal beats (SDNN) in ms.
- Proportion of NN50 divided by total number of normal-to-normal beats intervals (pNN50) (NN50 is the number of pairs of successive intervals between normal-to-normal beats that differ by more than 50 ms).

#### Driving performance in the simulator

Four driving variables were extracted from a standardized report generated by the simulator, based on the VirageSimulation algorithm:

- Percentage of time spent over the speed limit (Speeding);
- Amount of driving errors (Mistakes) including collisions with object/vehicle/pedestrian, respect of signalisation, sudden stop and inappropriate handing of the vehicle in turns as well as lane changes;
- General performance score (VSI Score) on 100 points resulting from the cumulative weight of six driving elements: steering control (15%), safety (30%), legality (30%), vehicle mobility (5%), road sharing (10%) and ecodriving (10%);
- Specific performance score from the six previous elements was calculated on 100 points: steering control (VSI Steering), safety (VSI Safety), legality (VSI Legality), vehicle mobility (VSI Mobility), road sharing (VSI Sharing) and ecodriving (VSI Ecodriving).

#### UFOV and crash risk prediction

Based on 4 measures obtained from the UFOV test [13]:

- Processing speed in milliseconds (ms) (subtest 1);
- Divided attention in ms (subtest 2);
- Selective attention in ms (subtest 3);
- From these measures, crash risk categories were obtained: 1) very low, 2) low, 3) moderate, 4) high and 5) very high.

### Statistical Analysis

For the control group, one-way repeated measure ANOVA (comparison between time points) was performed to measure the potential learning effect due to repeated exposure on the simulator and UFOV test. If the result was significant (*p* < 0.05) a Holm-Sidak post-hoc method was conducted.

For the experimental group, one-way repeated measures ANOVAs (comparison between time points) were performed to measure the effects of decreasing BAC on physiological response, driving behaviour and UFOV measures. When *p* value was significant (*p* < 0.05), a Holm-Sidak post-hoc method was conducted. Crash risk categories were compared using a Friedman repeated measures analysis of variance on ranks. When *p* value was significant (*p* < 0.05), a Dunnett’s post-hoc method was conducted. Spearman correlation coefficients were calculated to measure the relationships between driving performance and crash risk categories. Pearson correlation coefficients were completed to measure the relationships within and between driving performance and UFOV subtest performance. SigmaPlot (version 12.5; Systat Software inc., USA) and Microsoft Excel (version 14.0.7; Microsoft, USA) were used to conduct all analyses.

## Results

**Fig 1:**
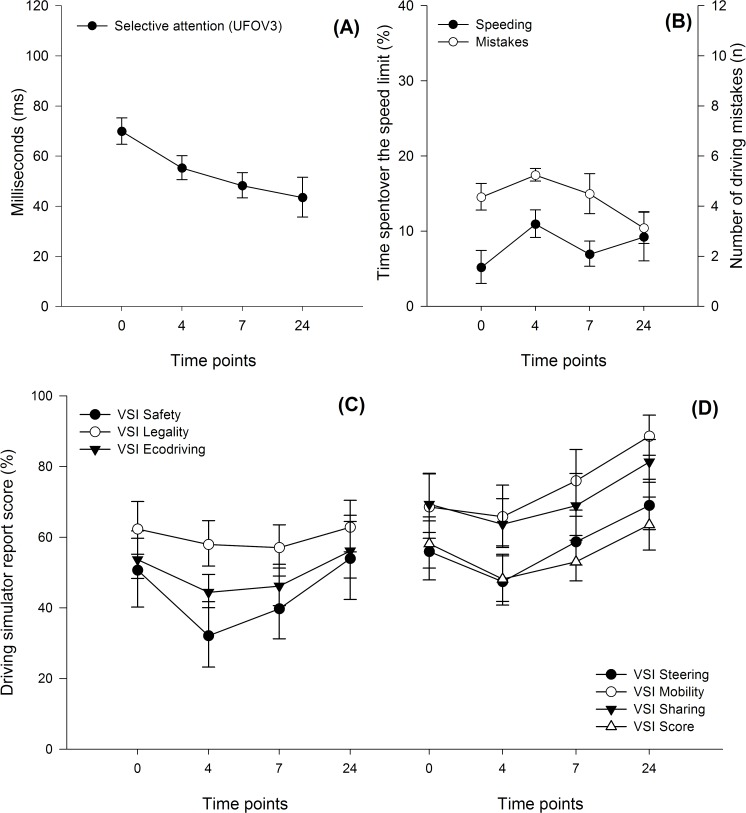
Timeline of driving variables and selective attention (useful field of view test) for the control group. (A) Selective attention scores (in milliseconds) for subtask 3 of useful field of view test; (B); Driving performance variables at the four assessed time points; (C) & (D) 7 driving simulator report variables divided into two graphs at four assessment time points.

For the control group, in all time points, results showed no learning effect from the driving simulator, but a learning effect for the UFOV was observed considering an improvement for the subtest three (selective attention) (Table 1). Graphical representation of these data can be visualized in Fig. 1.

**Table 1:** Driving performance, useful field of view test (UFOV) and physiological responses for the control group.

	Conditions (Time points)				*p* Value			
	0H	4H	7H	24H	ANOVA	Post-hoc		
						0H vs. 4H	0H vs. 7H	0H vs. 24H
**Driving performance**								
Speeding (%)	5.3 ± 6.2	11.0 ± 5.2	7.0 ± 4.8	9.3 ± 8.6	NS	—	—	—
Mistakes (n)	4.4 ± 1.5	5.3 ± 0.7	4.5 ± 2.3	3.1 ± 1.7	NS	—	—	—
**General**								
VSI Score (%)	58.5 ± 20.5	48.5 ± 18.8	53.4 ± 16.3	63.9 ± 19.8	NS	—	—	—
**Specific**								
VSI Steering (%)	56.3 ± 23.6	47.8 ± 19.6	59.0 ± 19.5	69.2 ± 18.9	NS	—	—	—
VSI Safety (%)	51.0 ± 30.5	32.5 ± 26.2	40.1 ± 25.2	54.3 ± 31.5	NS	—	—	—
VSI Legality (%)	62.6 ± 21.1	58.3 ± 18.2	57.4 ± 17.3	63.1 ± 19.3	NS	—	—	—
VSI Mobility (%)	68.9 ± 26.0	66.1 ± 24.3	76.3 ± 24.3	88.9 ± 15.0	NS	—	—	—
VSI Sharing (%)	69.6 ± 23.4	64.0 ± 19.6	69.3 ± 24.9	81.6 ± 16.0	NS	—	—	—
VSI Ecodriving (%)	54.0 ± 16.1	44.8 ± 13.3	46.5 ± 16.5	56.4 ± 21.2	NS	—	—	—
**UFOV**								
Processing speed (ms)	17 ± 0	17 ± 0	17 ± 0	17 ± 0	—	—	—	—
Divided attention (ms)	17 ± 0	17 ± 0	17 ± 0	17 ± 0	—	—	—	—
Selective attention (ms)	70.0 ± 14.9	55.4 ± 13.5	48.4 ± 14.3	43.6 ± 22.4	*	NS	*	*
Crash risk category	Very low	Very low	Very low	Very Low	—	—	—	—
**HRV**								
MHR (bpm)	75.7 ± 15.7	76.5 ± 14.0	76.4 ± 16.2	73.8 ± 13.6	NS	—	—	—
MeanNN (ms)	821.6 ± 163.0	808.4 ± 154.2	817.4 ± 172.8	837 ± 155.2	NS	—	—	—
SDNN (ms)	87.6 ± 38.3	75.4 ± 30.3	77.3 ± 32.8	85.4 ± 32.8	NS	—	—	—
pNN50 (%)	26.8 ± 25.9	26.0 ± 24.9	27.8 ± 27.9	31.2 ± 21.9	NS	—	—	—

Results presented are means and standard deviations (M ± SD); p Value from one-way repeated measure ANOVA and Holm-Sidak post-hoc test:

* p < 0.05

(—) non-assessed; (NS) non-significant. Results presented for crash risk category are the first on five level. (MHR) mean heart rate; (MeanNN) mean of NN intervals; (SDNN) standard deviation of NN intervals; (pNN50) proportion of NN50 divided by total number of NN intervals, (NN50) number of pairs of successive NN intervals that differ by more than 50ms.

**Fig 2:**
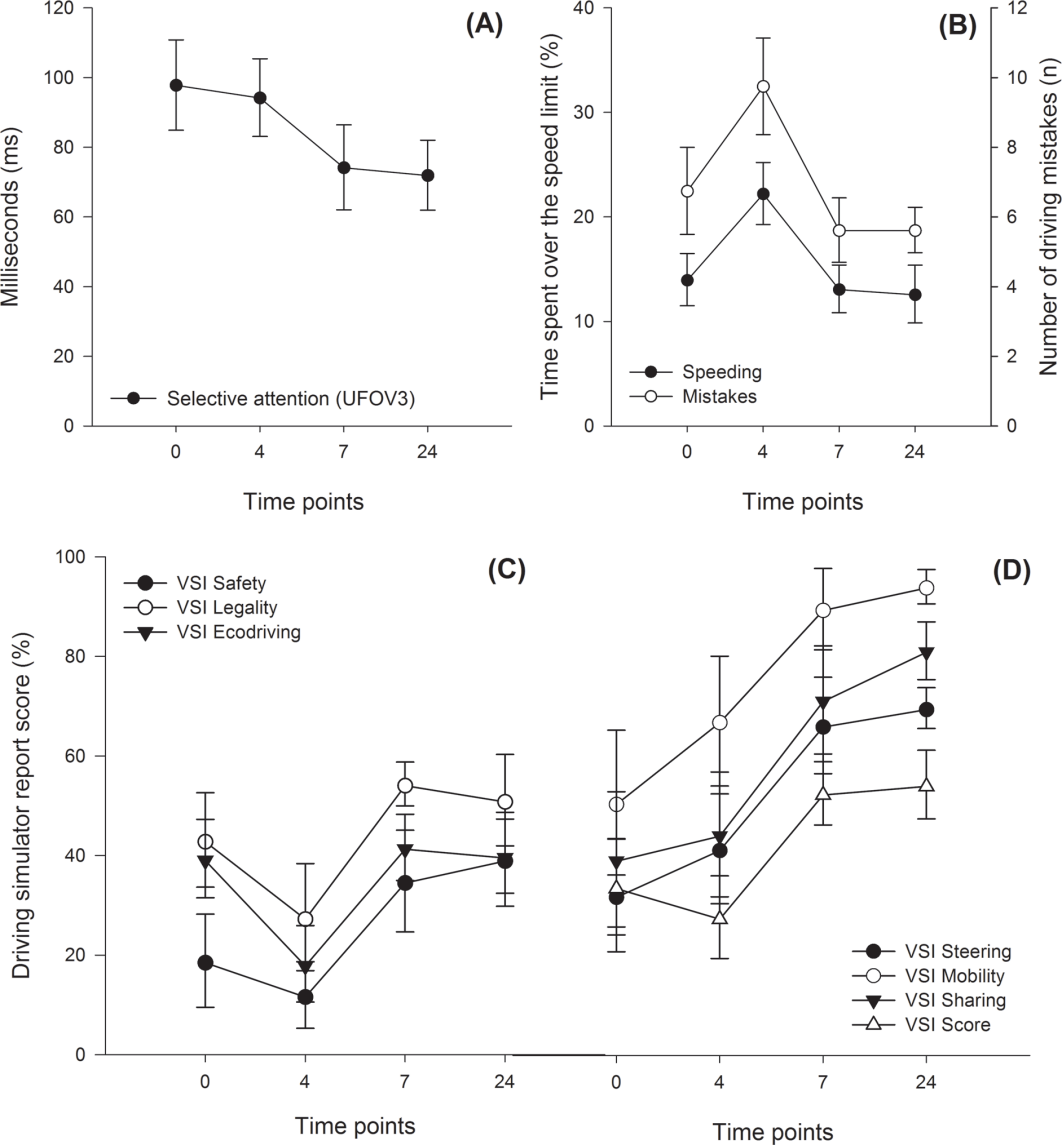
Timeline of driving variables and selective attention for the experimental group. (A) Selective attention scores (in milliseconds) for subtask 3 of useful field of view test; (B); Driving performance variables sensitive to moderate blood alcohol concentration (0.05–0.07 g/dL); (C) Driving simulator report variables sensitive to moderate blood alcohol concentration (0.05–0.07 g/dL); (D) Variables sensitive to driving simulator learning effect.

For the experimental group, physiological responses (i.e. changes in HRV) to alcohol were observed, as well as diminished driving performance, but crash risk did not change for all time points (Table 2). The summary of the repeated measures ANOVAs findings are presented in Table 2. Graphical representation of these data are presented in Fig. 2. Table 3 shows moderate correlations between driving variables. However, no correlations were observed between crash risk categories nor selective attention and driving variables.

**Table 2:** Driving performance, useful field of view test (UFOV) and physiological responses of post-acute alcohol consumption.

	Conditions (Time points)				*p* Value			
	Sober (0H)	Moderate BAC (4H)	Low BAC (7H)	Sober 24H	ANOVA	Post-hoc		
						0H vs. 4H	0H vs. 7H	0H vs. 24H
**Driving performance**								
Speeding (%)	14.0 ± 7.1	22.3 ± 8.4	13.1 ± 6.5	12.6 ± 7.8	**	*	NS	NS
Mistakes (n)	6.8 ± 3.5	9.8 ± 3.9	5.6 ± 2.6	5.6 ± 1.8	**	*	NS	NS
**General**								
VSI Score (%)	33.9 ± 27.3	27.8 ± 23.5	52.6 ± 18.0	54.4 ± 19.5	**	NS	*	*
**Specific**								
VSI Steering (%)	32.1 ± 32.0	41.5 ± 31.2	66.3 ± 27.4	69.8 ± 11.6	**	NS	**	**
VSI Safety (%)	18.9 ± 26.4	12.0 ± 18.9	34.9 ± 28.9	39.3 ± 26.7	*	NS	NS	NS
VSI Legality (%)	43.1 ± 26.8	27.6 ± 30.4	54.4 ± 12.5	51.1 ± 26.0	NS	—	—	—
VSI Mobility (%)	50.8 ± 41.2	67.1 ± 36.9	89.6 ± 23.1	94.1 ± 9.8	**	NS	**	**
VSI Sharing (%)	39.4 ± 38.4	44.4 ± 35.5	71.4 ± 30.7	81.3 ± 16.4	**	NS	*	**
VSI Ecodriving (%)	39.4 ± 22.3	18.3 ± 21.6	41.6 ± 18.8	39.9 ± 21.1	*	*	NS	NS
**UFOV**								
Processing speed (ms)	17 ± 0	17 ± 0	17 ± 0	17 ± 0	—	—	—	—
Divided attention (ms)	17 ± 0	24 ± 19.8	24.5 ± 21.2	17 ± 0	NS	—	—	—
Selective attention (ms)	97.9 ± 36.7	94.3 ± 31.6	74.3 ± 34.5	72.0 ± 28.4	*	NS	NS	NS
Crash risk category	Very low	Very low	Very low	Very Low	—	—	—	—
**HRV**								
MHR (bpm)	79.1 ± 12.0	93.4 ± 12.5	91.7 ± 10.1	79.0 ± 10.5	***	***	***	NS
MeanNN (ms)	774,9 ± 126.2	652.5 ± 85.3	660.7 ± 70.0	773.0 ± 113.9	***	***	***	NS
SDNN (ms)	75.9 ± 32.8	53.8 ± 21.1	53.1 ± 16.6	73.1 ± 21.2	**	**	*	NS
pNN50 (%)	22.7 ± 20.5	9.9 ± 16.8	9.3 ± 12.1	22.9 ± 16.6	*	*	*	NS

Results presented are means and standard deviations (M ± SD); p Value from one-way repeated measure ANOVA and Holm-Sidak post-hoc test:

* p < 0.05

** p < 0.01

*** p < 0.001

(—) non-assessed; (NS) non-significant. Results presented for crash risk category are the first on five level. (MHR) mean heart rate; (MeanNN) mean of NN intervals; (SDNN) standard deviation of NN intervals; (pNN50) proportion of NN50 divided by total number of NN intervals, (NN50) number of pairs of successive NN intervals that differ by more than 50ms.

**Table 3:** Correlations between driving performances, selective attention and crash risk categories for the experimental group.

	Mistakes	VSI score	Selective attention	Crash risk category
**Speeding**	0.63*	-0.70*	NS	NS
**Mistakes**	-	-0.71*	NS	NS
**VSI score**	-	-	NS	NS

Results present *r* value;

*p<0.05 from Pearson correlation. The association between crash risk category and drinving performances varaibles was measured by Spearman correlation; General driving performance score (VSI Score); (NS) non-significant

### Physiological alcohol response (HRV)

Under the influence of alcohol (moderate and low BAC), repeated measures ANOVA showed a higher MHR (*p* < 0.001). Lower MeanNN (*p* < 0.001), SDNN (*p* = 0.004) and pNN50 (*p* = 0.013) were also observed (Table 2). Post-hoc analysis indicated a higher MHR at moderate and low BAC (*p* = 0.039; *p* = 0.046 respectively). Additionally, the MeanNN, SDNN, and pNN50 were lower at moderate and low BAC, when compared to sober (*p* < 0.001; *p* = 0.010; *p* = 0.039, moderate BAC) (*p* < 0.001; *p* = 0.011; *p* = 0.046, low BAC). No significant differences were found for any HRV variables between sober and after 24 hours conditions.

### Driving performance and simulator learning effect

No learning effect was demonstrated by the driving simulator, as none of the driving performance variables were significantly different between the time points for the control group.

However, the group that consumed alcohol at moderate BAC exhibited decreased driving performance as they spent 8.3% more time over the speed limit (*p* = 0.011) and were implicated in 3 additional driving errors (*p* = 0.023) when compared to their sober drive (Table 2). A driving performance improvement at low BAC and after 24 hours, when compared to sober driving was observed for VSI Score (*p* = 0.034; *p* = 0.030), VSI Steering (*p* = 0.003; *p* = 0.002), VSI Mobility (*p* = 0.004; *p* = 0.002), VSI Sharing (*p* = 0.014; *p* = 0.002). No significant differences were found for VSI Legality variables at any conditions. Moreover, VSI Safety has a significant difference (*p* = 0.023), but post-hoc test shows no differences between sober and other time points. Significant relationships between Speeding and VSI Score (*r* = -0.70), Mistakes and VSI Score (*r* = -0.71) as well as Mistakes and Speeding (*r* = 0.63) were also observed and are presented in Table 2 for the group with alcohol.

### UFOV: learning effect, subtest measures and crash risk prediction

The control group obtained faster selective attention times at 7H (difference of mean = 21.4 ms; *p* = 0.033) and 24H (difference of mean = 26.4 ms; *p* = 0.014) time points, when compared to 0 H (Table 1). This shows a learning effect for this group in the third UFOV subtest. This group remained in the very low crash risk category throughout testing.

For the experimental group, no significant difference was found between the first and the second subtests (Table 2). However, the third subtest shows significant differences (*p* = 0.033). Over time points, we observe a decrease in selective attention time, however there are no significant differences when compared to sober (*p* = 0.732; *p* = 0.068; *p* = 0.064, respectively). Moreover, this group remained in the very low risk to crash category for all time points.

Additionally, there were no significant relationships between crash risk categories and driving variables. No significant relationships were found between selective attention and Speeding nor Mistakes (Table 3).

## Discussion

Our observations of four simulated driving sessions over 24 hours have shown no significant differences in driving performance in the control group. This allows us to rule out a learning effect from the driving simulator in young drivers. However, the measures of selective attention (UFOV, subtest three) were significantly faster at 7 hours and 24 hours. These results demonstrate a learning effect for this task. Other UFOV tasks were at a maximum throughout testing (17 ms).

The effects of alcohol were measured across three major aspects over various time points. First, physiological assessment by HRV was sensitive enough to measure different levels of BAC, as variables were only significantly affected at moderate (0.05–0.07 g/dL) and low BACs (0.01–0.04 g/dL). Moreover, after 24 hours post-acute consumption, HRV values were similar to the sober condition, showing that any physiological measurable alcohol effects are no longer present. Second, two driving variables were influenced by alcohol. Participants drove faster and made more driving errors at moderate BAC, when compared to their sober drive. No difference was found for these variables at low BAC and 24 hours. Nonetheless, other variables presented significant results at low BAC and at 24 hours, indicating an increased driving performance when compared to sober. These variables are VSI Score, VSI Steering, VSI Mobility and VSI Sharing. Third, the results of UFOV placed this group in the same crash risk category through all time points (i.e. very low risk). However, the subtest three shows a tendency of improvement at low BAC and 24 hours.

### Driving simulator: the effects of learning and alcohol expectancy

Even though the control group did not show a learning effect with the driving simulator, the alcohol group seems to have had one, given that variables were improved after 24 hours. The study conducted by Sahami et al. [16] demonstrated that although overall learning effects present large inter-individual differences, practice does in fact generally impact on the steering wheel and pedals control performance. The present study corroborates these findings as in addition to improvements in overall driving scores, differences were observed for VSI Score, VSI Steering, VSI Mobility and VSI Sharing (measures linked to steering and pedal control). Therefore, these variables should be considered when conducting drinking and driving simulator research in order to observe behavioural adaptations to the environment. However, taking into consideration that no learning effects were expected to be observed, another hypothesis may explain these disparities.

Oei & Morawaska [17] and Testa et al. [18] have observed behaviour changes in individuals anticipating alcohol consumption. These changes can be linked to the environment, the influence of peers and the alcohol expectancy effect (AEE). According to Oei & Morawaska [17], the AEE is the dominating factor. In addition, there is a social influence on AEE where individuals can find themselves distracted from their task [18]. Furthermore, past drinking experiences may modulate behavioural responses to alcohol. Therefore, the AEE could have interfered with driving performances between sober and moderate BAC. This may be explained by the fact that participants changed their behaviour before alcohol consumption (i.e. sober at 0 hour). Being accompanied by a friend could play into the social factor explained by Testa et al. [18]. Additionally, this interference may have had a negative impact on learning effects to simulated driving.

### UFOV learning effect

Learning effects for subtest three in the control group were observed. However, a tendency towards similar improvement for the alcohol group was present. Alcohol seems to have played a slowing factor in the learning effect for a selective attention task.

A few research teams studied the learning effect indirectly. Bentley et al. [19] assessed the test-retest reliability and the repeatability of the UFOV with young people. Significant improvements were observed between two UFOV tests administered 2 to 3 weeks apart. With different participants, they administered five tests in the same day and only observed significant improvements between the two first trials. Another research group, observed a retention time of 3 months, for young participants after 9 days of training (1 per day) [20], In the present study, we were able to see similar learning effects as both groups of participants were UFOV neophytes.

### Driving performance with decreasing BAC

Using data from traffic fatalities, French & Gumus [9] observed that young drivers with BACs below 0.08 g/dL are more prevalent in traffic collisions. To clarify these observations, the present study directly measured driving performance on a simulator a few hours after a social binge-drinking bout. Our results show an increased number of errors occurred at higher driving speeds while driving just under the legal BAC limit (moderate BAC), therefore increasing the risk of traffic collisions. These findings partially agree with those of Moskowitz & Florentino [21] and Schnabel [22], who found that both low and moderate BAC induced driving impairments. However, we were unable to demonstrate driving impairment at low BACs. Our results seem to corroborate Hegg-Deloye et al. [23] findings, which a low BAC is not enough to alter driving behaviours in a controlled driving environment. Methodologically, we observed that low BAC was achieved after a 7 hour wait from a binge-drinking bout, while their participants drank 1 to 2 beers in a 30 minute window to achieve the same level of intoxication.

McGwin et al. [24] have shown that crash involvement of younger drivers follows a trend for risk-taking behaviour and lack of driving skill. Mainly, a tendency for speeding and other risk taking behaviours may explain their implication in collisions. The results of the present study follow these same tendencies; under the influence of moderate BAC, younger drivers drove faster and showed an increased number of driving errors.

As discussed above, the AEE may have had an unexpected effect on driving performance in the alcohol consumption group. The AEE impacts a multitude of social, cognitive and motor behaviour. Burian et al. [25], have researched the effect of AEE on simulated driving performance. Risk-taking behaviour during a simulated driving task changed whether the participants were expecting alcohol or not. This finding may help in explaining the lack of expected differences between sober and moderate BAC in the present study.

### Crash risk prediction

The immediate effects of alcohol on higher-order cognitive functioning have been studied [26]. The UFOV, a test of visual processing, is one of the better known predictors of driving performance, usually used in older populations [12,13,27]. However, few studies have looked at its interaction with young adults, alcohol and driving. The present study shows that using five categories to predict the level of crash risk of a young individual is not sensitive enough. Furthermore, this classification system is unable to measure differences between different BAC at different time points in the same population. Moreover, when the results of the three subtests are analysed separately, we are able to observe that selective attention is the only measure presenting variablity. Yet, no differences were found at moderate BAC compare to sober. Dry et al. [26] observed no differences in selective attention for a moderate BAC, however high BAC decreased performance. In accordance with our results, a moderate BAC was not enough to decrease UFOV selective attention scores. This leads us to believe that the UFOV test, in younger populations, is mostly sensitive to large variations of BAC.

For other UFOV subtests, the lack of variability in processing speed and divided attention scores indicate that there may be a floor effect, demonstrating that these tasks are not adapted for young driver evaluation. Also, there was no relationships between UFOV measures, crash risk categories, and driving variables, further indicating that this tool is not able to predict driving risk of young drivers. This underscores the need to develop more sensitive and specific evaluation tools for this population.

### Practical implications

Our results demand attention to 2 key points in intervention plans targeting young drivers. First, during social events, it is not uncommon for young drivers to wait a few hours until they have reached a legal BAC before taking the wheel. However, our results clearly indicate that waiting a few hours (i.e. moderate, legal BAC) after acute consumption is not a safe strategy due to increased at-risk behaviour. Second, an increased amount of risk caused by the AEE in young drivers deserves further attention. While driving to and from a social gathering, young adults may go together, potentially mimicking driving under the influence behaviours and tend towards risky behaviours by peer pressure [28].

### Future research

Many drinking and driving research protocols tend to focus on post-consumption effects. In regards to our results, AEE may play a larger than expected role in driving sober with friends, to a social event. This avenue of drinking and driving research warrants more attention. Adding a social and pre-consumption aspect to current research protocols, we would be able to gather a more complete overview of drinking and driving in young adults.

### Limitations

For safety reasons, driving was performed on a driving simulator, therefore our results may not directly reflect real-world driving aptitudes. Moreover, during the adaptation period before assessment, simulator and UFOV exposure should be optimized in order to minimize any potential adaptation to the apparatus. In addition, possible drug usage by the participants was not controlled, nor drinker profile. Furthermore, driving history (i.e. driving frequency, years of driving experience, history of traffic violations and accidents) was not recorded. Because the study involved alcohol, the absence of a placebo condition in order to control for probable AEE could have impeded in the interpretation of data. Moreover, when conducting studies with young individuals (i.e. undergraduate students), a priming effect to alcohol could have played a part in potential confounding results. Since these individuals were shown the bottle of alcohol and knew the quantity that they would have to drink (i.e. priming effect), this may be a partial explanation for the potential AEE observed. Nonetheless, the present study was a pilot study regarding the effects of BAC on driving performance in undergraduate students. We focused on a condensed number of variables, therefore statistical power was low, limiting between group analysis.

## Conclusion

In order to better reflect the practices of undergraduate students after acute alcohol consumption, our methodology explored descending limb of blood alcohol concentrations. Our findings add understanding of the behaviours linked to the overrepresentation of young drivers under the influence of alcohol in fatality statistics, as moderate blood alcohol concentrations increased driving speeds and the number of errors commited by the drivers. Also, the useful field of view test was not sensitive enough to measure performance differences due to alcohol consumption. This tool is not a good predictor of crash risk in young adults and in young adults under the influence of alcohol. Moreover, when driving to go to a social event young drivers may unknowingly be at risk. The alcohol expectency effect may aid in the explanation of the disparity of differences between sober and moderate blood alcohol concentrations demonstrated by our participants. This avenue of drinking and driving research warrants further understanding.

## Supporting Information

S1 Data Demographic, driving, crash risk and physiological responses data for each participant, group and time points. Format is in 22 columns and 65 lines (semi-colons for separator and points for decimal): Column 1: (ID) Identification number of participant; Column 2: (Group) Control or Experimental group; Column 3: (TimePoints) 0 hours, 4 hours, 7 hours and 24 hours; Column 4: (Gender) Female (F) or Male (M); Column 5: (Age) Age in years; Column 6: (Speeding) Percentage of time spent over the speed limit; Column 7: (Mistakes) Number of driving mistakes; Column 8: (VSI_SCORE) General performance score on 100 points from the driving simulator report; Column 9: (VSI_Control) Steering control performance score on 100 points from the driving simulator report; Column 10: (VSI_Safety) Safety performance score on 100 points from the driving simulator report; Column 11: (VSI_Legality) Legality performance score on 100 points from the driving simulator report; Column 12: (VSI_Mobility) Vehicle mobilty performance score on 100 points from the driving simulator report; Column 13: (VSI_Sharing) Road sharing performance score on 100 points from the driving simulator report; Column 14: (VSI_Ecodriving) Ecodriving performance score on 100 points from the driving simulator report; Column 15: (UFOV_Categories) Crash risk categories (i.e. very low, low, moderate, high and very high); Column 16: (UFOV1) Processing speed in milliseconds (subtest 1); Column 17: (UFOV2) Divided attention in milliseconds (subtest 2); Column 18: (UFOV3) Selective attention in milliseconds (subtest 3); Column 19: (MHR) Mean heart rate in beat per minutes; Column 20: (MeanNN) Mean time intervals between normal-to-normal beats in milliseconds; Column 21: (SDNN) Standard deviation time intervals between normal-to-normal beats in milliseconds; Column 22: (pNN50) Proportion of NN50 divided by total number of normal-to-normal beats intervals (NN50 is the number of pairs of successive intervals between normal-to-normal beats that differ by more than 50 milliseconds).
(CSV)
